# Subtypes of borderline personality disorder patients: a cluster-analytic approach

**DOI:** 10.1186/s40479-017-0066-4

**Published:** 2017-07-03

**Authors:** Maaike L. Smits, Dine J. Feenstra, Dawn L. Bales, Jasmijn de Vos, Zwaan Lucas, Roel Verheul, Patrick Luyten

**Affiliations:** 1 Viersprong Institute for Studies on Personality Disorders, Halsteren, The Netherlands; 2 Expertisecentrum MBT-NL, Bergen op Zoom, The Netherlands; 3 Netherlands Psychoanalytic Institute, Amsterdam, The Netherlands; 4 Lentis, Groningen, The Netherlands; 5 Department of Clinical Psychology, University of Amsterdam, Amsterdam, The Netherlands; 6 Faculty of Psychology and Educational Sciences, University of Leuven, Leuven, Belgium; 7 Research Department of Clinical, Educational and Health Psychology, University College London, London, UK

**Keywords:** Borderline personality disorder, Cluster analysis, Subtypes, Comorbidity, Personality dimensions

## Abstract

**Background:**

The borderline personality disorder (BPD) population is notably heterogeneous, and this has potentially important implications for intervention. Identifying distinct subtypes of patients may represent a first step in identifying which treatments work best for which individuals.

**Methods:**

A cluster-analysis on dimensional personality disorder (PD) features, as assessed with the SCID-II, was performed on a sample of carefully screened BPD patients (*N* = 187) referred for mentalization-based treatment. The optimal cluster solution was determined using multiple indices of fit. The validity of the clusters was explored by investigating their relationship with borderline pathology, symptom severity, interpersonal problems, quality of life, personality functioning, attachment, and trauma history, in addition to demographic and clinical features.

**Results:**

A three-cluster solution was retained, which identified three clusters of BPD patients with distinct profiles. The largest cluster (*n* = 145) consisted of patients characterized by “core BPD” features, without marked elevations on other PD dimensions. A second “Extravert/externalizing” cluster of patients (*n* = 27) was characterized by high levels of histrionic, narcissistic, and antisocial features. A third, smaller “Schizotypal/paranoid” cluster (*n* = 15) consisted of patients with marked schizotypal and paranoid features. Patients in these clusters showed theoretically meaningful differences in terms of demographic and clinical features.

**Conclusions:**

Three meaningful subtypes of BPD patients were identified with distinct profiles. Differences were small, even when controlling for severity of PD pathology, suggesting a strong common factor underlying BPD. These results may represent a stepping stone toward research with larger samples aimed at replicating the findings and investigating differential trajectories of change, treatment outcomes, and treatment approaches for these subtypes.

**Trial registration:**

The study was retrospectively registered 16 April 2010 in the *Nederlands Trial Register*, no. NTR2292.

## Background

Borderline personality disorder (BPD) is among the most prevalent personality disorders [1]. BPD is associated with a high disease burden in terms of high levels of psychiatric comorbidity, low quality of life, high levels of acting out, and a high lifetime risk of completed suicide, as well as high societal costs [2].

The BPD population is notably heterogeneous from a descriptive and theoretical perspective. Two hundred fifty-six possible combinations of criteria may yield the same diagnosis. Hence, two patients with a diagnosis of BPD may have only one diagnostic criterion in common [3]. Moreover, the high prevalence of comorbid pathology amongst patients with BPD is widely recognized [4]. Therefore, large variation in expression of BPD pathology is apparent in clinical practice. Heterogeneity in the BPD population poses challenges in clinical practice with regard to treatment approach. Although it has been previously noted that, given the heterogeneity of the disorder, it is unlikely that any so-called “one size fits all” treatment could be identified [5], this heterogeneity has been insufficiently taken into account in existing evidence-based treatments [6, 7]. Empirical evidence for a variety of treatments for BPD, such as Transference-Focused Psychotherapy; Systems Training for Emotional Predictability and Problem Solving, Dialectical Behavior Therapy, Schema-Focused Therapy, and Mentalization-Based Treatment is accumulating [8–10]. However, interpretation of treatment outcome in these studies is hampered by the fact that there may be substantial differences in outcome for different types of BPD patients. Related research on possible differences in response and trajectories of change is also hampered by the relative dearth of research on different subtypes of BPD patients [11]. Hence, in order to address the growing empirical and clinical need to identify which treatments work best for which patients, it is important to identify patient features that may be associated with differential treatment outcome and different trajectories of change. The need to identify meaningful subtypes has been equally stressed in research on developmental pathways involved in BPD (i.e. [4]). Our understanding of the etiology of BPD is currently hampered because of likely differences in etiological pathways toward different phenotypes of BPD [12]. The identification of meaningful subtypes is also important given the growing interest in early detection and intervention in clinical staging models of BPD [13]. This may ultimately lead to improved theoretical frameworks and treatments that are tailored to the specific features and stage of problems presented by a particular BPD patient.

Improved understanding of subtypes may promote refinement of treatment models differentially targeted at salient patient characteristics, thus optimizing the effectiveness of these programs [14]. Several studies have addressed this issue, but results have been quite inconclusive.

### The search for subtypes of BPD patients

Existing research aimed at identifying clinically meaningful subtypes of BPD has, broadly speaking, taken either a variable-centered or a person-centered approach. The former approach has mainly relied on factor analysis, the latter on latent class analysis, cluster analysis, and finite mixture modeling. Variable-centered approaches aim to reduce the wide variety of BPD criteria into a smaller number of underlying dimensions. These studies have typically found evidence for two- to four-factor solutions, encompassing factors such as interpersonal/relational and identity stability; impulsivity and affective instability, consisting of various constellations of the nine BPD criteria [15–17]. Results have been quite inconsistent, which may be due to differences in samples and measures of BPD used [18]. Moreover, correlations among the factors are typically very high, leading some authors to conclude that a more parsimonious one-factor model may fit the data best [19–24]. Elaborating on this knowledge, it has been suggested that BPD may be most adequately described by a one-dimensional model, with factors representing varying degrees of severity on the underlying continuum [21, 24]. Taking into account the evidence demonstrating BPD to be a unidimensional construct, the additional value of investigating the differentiating multiple-factor structures has been noted to be more useful to understand BPD comorbidity and to plan treatment [25].

Variable-based approaches, however, do not allow individual patients to be sorted into meaningful subtypes, and consequently are somewhat limited in their ability to address the question of heterogeneity in BPD [14]. Because individuals may show meaningful combinations of the identified underlying factors of the BPD construct [26], a person-centered approach may be more suitable. Studies comparing both approaches have generally reported evidence for one underlying dimension, with differentiated subtypes [22, 24]. Several studies in samples of clinical (inpatient and outpatient) and nonclinical participants using latent class analysis found clusters that differentiated between (a) individuals with few or no BPD criteria or low likelihood of BPD pathology and (b) individuals with a high number of BPD criteria or high likelihood of BPD pathology [19, 21], thus reflecting differences in the presence and severity of BPD pathology. This has led several authors to stress the need to move beyond differences in terms of severity and broaden the scope of research to features external to diagnostic criteria, in the search for the existence of qualitatively different subtypes among BPD patients [18, 19, 21].

Studies in this area are few, and to date have suggested the existence of two to four subtypes of BPD patients. Leihener et al. [12], for example, found two distinct subtypes based on interpersonal functioning, labeled *autonomous* and *dependent*. Salzer et al. [27] differentiated five subtypes based on their characteristic interpersonal patterns: *vindictive*, *moderate submissive*, *nonassertive*, *exploitable*, and *socially avoidant*. Zittel, Conklin, Bradley, and Westen [28] identified three subtypes—defined as *internalizing dysregulated*, *externalizing/dysregulated*, and *histrionic-impulsive*—based on a contrast analysis of clinician-rated affect experience and affect regulation. Yet, in an adolescent population, the same group of authors identified four subtypes: *high-functioning internalizing*, *histrionic*, *depressive internalizing*, and *angry externalizing* [29]. Digre, Reece, Johnson, and Thomas [30] found three subtypes by means of a two-step cluster analysis on demographic, clinical and psychological variables (i.e., age, comorbid diagnosis, coping strategies, suicide attempts and self-harm), which were labeled *withdrawn-internalizing*, *severely disturbed-internalizing*, and *anxious-externalizing*. Lenzenweger et al. [14] performed a theory-based finite mixture modeling analysis, which revealed three phenotypically distinct subtypes of patients, in line with the work of Kernberg and colleagues [31]: the first group was characterized by low levels of antisocial, paranoid, and aggressive features; the second by elevated paranoid features; and the third by elevated antisocial and aggressive features. Critchfield, Clarkin Levy, and Kernberg [32] used the same sample of patients, but, by means of Q-factor analysis based on co-occurring PD criteria, they found three subtypes of BPD patients: those with co-occurring cluster A PD traits (elevated schizotypal and paranoid features), those with cluster B PD traits (elevated narcissistic and histrionic features), and those with cluster C PD traits (elevated avoidant and obsessive-compulsive features). Hallquist and Pilkonis [18], by means of finite mixture modeling, found four subtypes that differed in terms of anger/aggressiveness/antisocial behavior and mistrustfulness: an *angry-aggressive* type with high levels of aggression, antisocial behavior, and dysfunctional bids to maintain interpersonal relationships; an a*ngry/mistrustful* type, characterized by considerable concerns about being harmed or exploited in relationships, alongside inappropriate anger; a *poor identity/low anger* type with poor sense of self and self-injurious behaviors, but low aggressiveness; and a *prototypical* type with moderate levels of anger but low levels of aggression, antisocial behavior and mistrustfulness. Although there is overlap between the subtypes that have been found in previous studies, no clear consensus has yet been reached on the identification of meaningful subtypes of BPD, resulting at least in part from the various different theoretical and methodological approaches that have been used in defining subtypes. Some studies have used a purely data driven approach, while others have adopted a theory-based approach, leading to multiple categorizations that are difficult to compare with one another. A major limitation of current research in this area is that the thus identified subtypes are often difficult to identify in clinical practice, which limits practical usability with regard to treatment selection or empirical research on treatment outcome. Further reseach on potential subtypes in BPD is therefore needed in a manner that facilitates the applicability of findings in both clinical and empirical practice.

## The present study

In response to the call for more empirical studies that are based on features of BPD [18], we therefore set out to identify subtypes of BPD patients based on information that is commonly available in clinical practice. The present study used a person-centered cluster-analytic approach to identify clusters of BPD patients based on comorbid PD dimensions, building on the study of Critchfield et al. [32]. Based on these commonly available patient characteristics that are often used for treatment selection, we explore whether there are meaningful subgroups that differ based on their PD profiles. The clusters were then validated by investigating their relationship with several domains that are both theoretically and clinically associated with BPD pathology in order to promote the recognizability and applicability of the subtypes in clinical practice. Validation measures included (severity) of borderline pathology, symptom severity, interpersonal problems, quality of life, personality functioning, attachment, and trauma history, in addition to demographic and clinical features. Finally, because a general severity dimension was found to obfuscate attempts to identify meaningful subtypes of BPD patient in earlier efforts [22, 24], we controlled for overall PD severity in all analyses.

## Methods

### Participants and procedures

Participants were 187 outpatients participating in a multicenter randomized controlled trial on the (cost-) effectiveness of day-hospital versus intensive outpatient MBT [2]. Participants were included between March 2009 and July 2014. Patients were referred for MBT at three mental health-care institutions in the Netherlands. All patients underwent a detailed screening and assessment, including semi-structured interviews (described later) to assess axis-I disorders and PDs. Patients were given both verbal and written information on the study, and gave written consent to participate. The study was approved by the Medical Ethics Committee of the Erasmus Medical Center, Rotterdam, the Netherlands. Data were obtained from all screened patients before patients were randomized to either MBT intervention.

Inclusion criteria for this study were having a formal diagnosis of BPD, being 18 years of age or older, and having adequate mastery of the Dutch language. Exclusion criteria were very minimal, comprising diagnosis of an autism spectrum disorder, chronic psychotic disorder, or organic brain disorder that might interfere significantly with the ability to mentalize, and intellectual impairment (IQ < 80). Hence, patients with marked substance abuse or antisocial features were eligible for inclusion in the study.

A total of 226 patients met the inclusion criteria. Thirty-nine patients were excluded because of missing data on the variables used in the cluster analysis (*n* = 20) or because they were extreme outliers, defined as having a score on the input dimension that deviated more than 3.29 standard deviations (*SD*) from the sample mean on the input variables (*n* = 19), leaving 187 patients for the current study. At baseline, these 187 patients had a mean age of 29.1 years (*SD* 8.7, range 18–56). The majority of patients (*n* = 164, 88%) were female. Mean scores on the validation measures for the total sample are presented alongside the cluster means in Table 3.

### Input clustering measures

#### Personality disorder features dimensional scores

PDs were assessed using the Structured Clinical Interview for DSM-IV Axis II Personality Disorders (SCID-II; [33, 34]). PD criteria were scored if they were pathological, persistent, and pervasive. Features of the PDs can be scored as 1 (*absent*), 2 (*uncertain*), or 3 (*positive*). Dimensional scores on all 10 PDs were computed by means of the sum of scores on all criteria of the PD. Interviewers were MSc-level psychologists or MSc students who were supervised by an experienced mental health-care psychologist and trained in the SCID-I (see below) and SCID-II by an expert trainer. Previous research has shown that both the original SCID-II and the Dutch version have good inter-rater and test–retest inter-rater reliability [35–37].

### Demographic and clinical features

#### Axis-I disorders

Axis-I disorders^1^ were assessed using the Structured Clinical Interview for DSM-IV Axis I Disorders (SCID-I; [38, 39]). The SCID-I has good inter-rater reliability (*κ* = .85), especially when interviewers receive training as in the present study [40].

### Validation measures

#### Borderline symptomatology and severity

Borderline symptomatology and severity was assessed by means of the Dutch version of the Personality Assessment Inventory borderline features scale (PAI-BOR; [41]). The PAI-BOR is a subscale of the Personality Assessment Inventory [42] and consists of four subscales (each containing six items), which reflect four characteristics of BPD – Affective Instability, Identity Problems, Negative Relationships, and Self-Harm – each with a score range of 0–18, and a total score range of 0–72. Both internal consistency of the total score (Cronbach’s α = .81) and subdomains (Cronbach’s α range .52–.69), and 6-month test–retest correlation for the sum score (Pearson’s *r* .78) and the subdomains (Pearson’s *r* range .60–.75) of the Dutch PAI-BOR are good [41]. Internal consistencies in the current sample were consistent with these estimates, with Cronbach’s α = .81 for the total score and ranging from .52 to.79 for the subdomains.

#### Symptomatic severity

General psychopathological symptoms were assessed with the Dutch version of the Brief Symptom Inventory (BSI; [43, 44]). The 53-item BSI is the short version of the Symptom Checklist-90-R [45, 46]. The Global Severity Index, with a score range of 0–4, was used as a global index of symptom distress. The reliability of the Dutch version of the BSI is good (Cronbach’s α ranging from .71 to .88, test–retest reliability *r* = .71–.89; [43]). Internal consistency in the current sample was also high (Cronbach’s α = .97).

#### Social and interpersonal functioning

Social and interpersonal functioning was assessed by a Dutch version of the Inventory of Interpersonal Problems, using either the 32-item or the 64-item version (IIP; [47, 48]). The IIP is a self-report measure assessing eight dimensions of interpersonal problems: Domineering/Controlling, Vindictive/Self-Centred, Cold/Distant, Socially Inhibited, Non-Assertive, Overly Accommodating, Self-Sacrificing, and Intrusive/Needy, with subscale scores ranging from 0 to 32 and a total score range of 0–256. The reliability of the Dutch IIP-64 (Cronbach’s α range .73–.85 for subscales and .93–.94 for the total score; [47, 48]) and original IIP-32 (Cronbach’s α range .68–.88 for subscales and .73–.85 for the total score; [49]) are good. In the current sample Cronbach’s α was high for the total score for both the 64-item and 32-item version; α = .94 and α = .81 respectivly. Likewise, for the subdomains internal consistency was sufficient, ranging from .66–.86 for the 64-item version, but somewhat lower for some subscales of the 32-item version (Chronbach’s α range from .32 to .81).

#### Quality of life

Quality of life was measured using the EuroQol EQ-5D-3 L [50]. This self-report questionnaire assesses health problems on five dimensions: mobility, self-care, usual activities, pain/discomfort, and anxiety/depression. The dimensions can be summarized into a “value” ranging from −1 to 1, based on the preferences of the general public. Also, respondents mark their current health on a vertical visual analogue scale (VAS), ranging from 0 (worst imaginable health) to 100 (best imaginable health). The reliability of the EQ-5D-3 L has been found to be acceptable [51]. Internal consistency was sufficient within the current sample (Chronbach’s α = .60).

#### Personality functioning

Personality functioning was assessed using the Severity Indices of Personality Problems (SIPP; [52]). Either the 60-item (SIPP-SF) or the 118-item (SIPP-118) version was used. The SIPP is a dimensional self-report measure assessing the severity of the changeable components of personality pathology. Higher scores relate to more adaptive personality functioning. In both versions, five higher-order domains are computed: Self-Control, Identity Integration, Responsibility, Relational Capacities, and Social Concordance, with score range of 1–4. Both the SIPP-118 (Cronbach’s α range .69–.84) and SIPP-SF have good psychometric properties [53] and internal consistency within the current sample was high for all domains for both the SIPP-118 (Cronbach’s α range .80–.88) and SIPP-SF (Cronbach’s α range .79–.88).

#### Attachment dimensions

The Experiences in Close Relationships questionnaire (ECR; [54]), was used to assess attachment avoidance and attachment anxiety. Subscale scores range from 1 to 7. The Dutch version of the ECR was found to be a valid measure with good internal (Cronbach’s α range .86–.93) and external validity [55]. Internal consistency for the current sample was high for both the attachment avoidance (Cronbach’s α = .94) and anxiety subscale (Cronbach’s α = .90).

#### Trauma

The prevalence of trauma in childhood was measured by means of a Dutch translation of the short form of the retrospective self-report Childhood Trauma Questionnaire (CTQ; [56]), which measures five categories of childhood trauma experience: emotional, physical, and sexual abuse, and emotional and physical neglect. Subscale scores range from 5 to 25 and the total score from 25 to 125. Both the original CTQ version (Cronbach’s α range .61–.95; [56, 57]) and the Dutch translation (Cronbach’s α range .63–.95; [58]) have adequate psychometric properties. Internal consistencies in the current sample were consistent with these estimates, with Cronbach’s α = .93 for the total score and ranging from .63 to .91 for the subdomains.

### Statistical analysis

All analyses were performed in SPSS version 23.0. The 10 PD dimensions served as input variables for the cluster analysis. Since the primary interest lay in the patterning across PD features, as opposed to identifying an overall severity level, all dimensional scores were adjusted for the overall severity of personality pathology (i.e., each cases' own mean score of PD features on the SCID-II), thereby eliminating within each person the influence of their overall severity on the PD profile. A two-phased clustering procedure was used following recent state-of-the art recommendations, described in detail in Gore [59]. The first step involved a hierarchical cluster analysis, by means of Ward’s method with squared Euclidean distances [60]. In the second step, the cluster-center means extracted through this hierarchical analysis were used as non-random starting points in a *k*-means cluster analysis [61]. This iterative procedure solves a major shortcoming of the hierarchical method, namely, that once a case is assigned to a cluster; it cannot be reassigned to another cluster in a subsequent stage. In the *k*-means clustering procedure the within-cluster variance on criterion variables is minimized, while differences between clusters are maximized, allowing reassignment of cases to a better fitting cluster, thus optimizing cluster membership [59]. Hence, the hierarchical cluster analysis based on dimensional scores on the PD dimensions was used to define clusters with distinct meaningful and coherent profiles representing different BPD subtypes. Subsequently, k-means clustering was used to assign individuals to their best fitting-cluster. This two-phased procedure that starts with a decision on the number of clusters, was repeated for the assumption of a 2-, 3-, 4-, 5- and 6 cluster solution. Different cluster solutions were compared with regard to the proportion of variance in the 10 input PD dimensions that was explained by the cluster solution (multivariate *R* ^*2*^; 1–Wilks’ lambda(Λ)) and a more conservative measure of the proportion of the variance that was accounted for by the cluster solution, taking into account the error factor in the analysis (partial ƞ^2^). The fit of the cluster solutions was also compared based on multiple information criteria: the Akaike Information Criterion [62], Schwarz’s Bayesian Information Criterion [63], Calinski-Harbasz Index [64], and Silhouettes [65]. Based on explanatory power, fit indices, parsimony, and interpretability, the best fitting model cluster solution was determined.

Kendall’s tau (**τ**) was used to investigate the relationships between the input dimensions. The clusters were then compared on the input dimensions by means of multivariate analysis of variance (MANOVA) with Games-Howell post-hoc comparisons. Because the clusters were defined using *z*-standardized scores, the cluster means are deviation scores from the total sample mean, with *M* = 0 and *SD* = 1. Thus, each cluster’s mean *z*-score indicated how far the cluster deviated from the total sample mean score (0) and from the means of the other clusters. [66, 67]. Discriminant analysis was used to investigate the dimensions underlying and accounting for the distinct clusters.

Finally, clusters were compared on external validation measures by means of chi-square tests or (M)ANOVA with Games-Howell post-hoc test, as appropriate. In case of violation of assumption of expected frequencies, Fisher’s exact test was used (chi-square test), and in case of violation of the assumption of equality of variances Welch’s *F* statistic was used (ANOVA). Effects sizes (ES) for the external validation measures were computed in the same manner as described above. As a result of missing data, sample sizes differ for each (M)ANOVA per instrument.

## Results

### Sample characteristics

Of the 187 patients included in the study, 80% (*n* = 149) had at least one axis-I disorder (range 0–6). Mood disorders were most frequently diagnosed (*n* = 100, 54%), followed by anxiety disorders (*n* = 75, 40%), substance use disorders (*n* = 54, 29%), and eating disorders (*n* = 49, 26%). About one third of patients was diagnosed with more than one PD (*n* = 58, 31%). Besides BPD, avoidant PD was most prevalent (*n* = 18, 10%). Cluster C PD traits were the most prevalent comorbid PD traits (*n* = 131, 72%). Of all patients, 53% (*n* = 100) had at least one avoidant PD feature, 34% (*n* = 63) at least one dependent PD feature, and 32% (*n* = 60) at least one obsessive-compulsive PD feature. For cluster B features, 12% (*n* = 23) of patients had at least one narcissistic PD feature, 12% (*n* = 22) at least one antisocial PD feature, and 6% (*n* = 12) at least one histrionic PD feature. Cluster A features were least prevalent; although 18% (*n* = 34) had at least one paranoid PD feature, only 6% (*n* = 12) had at least one schizotypal PD feature, and only one patient had a schizoid PD feature.

### Subtypes of BPD patients based on two-phase cluster analysis

Significant correlations between several of the PD dimensions were found, even after correcting for severity, ranging from τ = −.32 to τ = .74.^2^ Inspection of the percentage of variance in the personality dimensions that was accounted for by the cluster solution (multivariate *R* ^*2*^) revealed that the two-factor solution explained 81.0% of the total variance. A three-cluster solution explained 97.3% of the variance. The improvement in explained variance was very small for the four-, five-, and six-cluster solutions (99.1, 99.7, and 99.9%, respectively), suggesting that a three-factor solution was optimal in terms of parsimoniousness and explained variance. This assumption was confirmed when considering the more conservative measure of the proportion of variance accounted for by the cluster solution, by adding the influence of an error factor (partial ƞ^2^). Partial ƞ^2^ was also highest for the three-cluster solution (.84) compared with the two- to five-cluster solutions (respectively, .81, .79, .77, and .76). A six-cluster solution consisted of clusters containing very few patients (smallest cluster *n* = 3) and could not be meaningfully interpreted. Exploration of fit criteria for the two-, three-, four-, and five-cluster solutions showed inconsistent results (see Table 1). Because of parsimony and interpretability, the three-cluster model was retained.

**Table 1:** Fit indices of optimal cluster solution

Cluster solution	AIC	BIC	CH	S
Two clusters	1144.66	1273.91	24.58	.325^a^
Three clusters	1068.20	1262.06^a^	25.24	.313
Four clusters	1043.13	1301.62	24.95	.309
Five clusters	971.74^a^	1294.85	28.15^a^	.248

*AIC* Akaike Information Criterion, *BIC* Schwarz’s Bayesian Information Criterion, *CH* Calinski-Harabasz Index, and *S* Silhouettes

^a^Optimal fit according to this criterion. A better fit of the cluster solution to the data is indicated by higher CH and S scores, and lower AIC and BIC scores

**Fig. 1:**
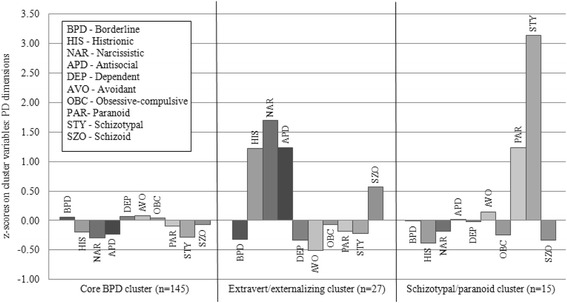
Z-scores on personality dimensions for the final 3-cluster solution. Z-scores below 0 represent lower and above 0 higher scores compared to the total sample mean

Figure 1 illustrates the final cluster solution. Cluster 1 was the largest cluster, consisting of 76% of the sample (*n* = 145). Patients in this cluster showed the highest relative levels of BPD features compared with the two other clusters and no marked elevations on the other dimensions. We therefore labeled this cluster “*Core BPD*”. The second cluster consisted of a smaller number of patients (14%, *n* = 27). We labeled this cluster “*Extravert/externalizing*,” because an outward-oriented/externalizing attitude seemed to be a common denominator in the narcissistic, antisocial, and histrionic PD dimensions on which the cluster differentiated from the other two clusters. The smallest cluster consisted of 8% of the patients (*n* = 15), and was labeled “*Schizotypal/paranoid*” because of the elevated levels on these PD dimensions.

A MANOVA showed a significant difference between the three clusters on the clustering dimensions, Λ = .027, *F*(20, 350) = 88.429, *p* < .001. Clusters differed significantly on all PD dimensions except for the BPD dimension itself, and the dependent and obsessive-compulsive PD dimension (see Table 2). The *Schizotypal/paranoid* cluster differed markedly from the other clusters, as expressed in very high ES on the schizotypal and paranoid PD dimensions. The *Extravert/externalizing* cluster differed from the other clusters, as expressed in very high ES on the narcissistic, antisocial, and histrionic PD dimension (see also Fig. 1). Compared with the other two clusters, the *Extravert/externalizing* cluster scored very low on the avoidant PD dimension, resulting in a significant difference between the *Core BPD* and *Extravert/externalizing* cluster.

**Table 2:** Differences on personality dimensions for the three-cluster solution

PD dimension	Core BPD		Extravert/externalizing		Schizotypal/Paranoid		*F*	Games-Howell Post-hoc comparison and *d*
	*M*	*SD*	*M*	*SD*	*M*	*SD*		
Borderline	.06	1.01	−.32	.94	−.01	1.01	1.618	
Histrionic	−.19	.50	1.22	1.98	−.38	.14	31.715***	2 > 1,3 (*d* = 1.41; 1.60); 1 > 3 (*d* = .19)
Narcissistic	−.30	.41	1.69	1.58	−.18	.63	87.427***	2 > 1,3 (*d* = 1.99; 1.87)
Antisocial	−.23	.44	1.24	1.87	.01	1.12	32.993***	2 > 1,3 (*d* = 1.47; 1.23)
Dependent	.06	1.04	−.33	.79	−.02	.88	1.837	
Avoidant	.08	1.02	−.51	.61	.15	1.13	4.283*	2 < 1 (*d* = .59)
Obsessive-compulsive	.04	1.02	−.07	.90	−.25	1.00	.637	
Paranoid	−.10	.95	−.18	.59	1.24	1.27	14.413***	3 > 1,2 (*d* = 1.34; 1.42)
Schizotypal	−.28	.26	−.22	.43	3.13	.90	573.271***	3 > 1,2 (*d* = 3.41; 3.35)
Schizoid	−.07	.60	.57	2.13	−.34	.61	5.882**	*ns*

The last column summarizes the significant post-hoc comparisons with corresponding effect sizes (Cohen’s *d*) between the cluster means per dimension; > corresponds to a higher dimensional score and < to a lower dimensional score. *p < .05, **p < .01, ***p < .001

The MANOVA was followed up with a discriminant analysis, which revealed two discriminant functions. The first explained 68.1% of the variance, canonical *R* ^*2*^ = .88, and the second 31.9%, canonical *R* ^*2*^ = .76. These discriminant functions significantly differentiated between the clusters, both in combination (Λ = .027, χ^2^(20) = 646.41, *p* < .001) and when the first function was removed (Λ = .226, χ^2^(9) = 266.66, *p* < .001). To make interpretation easier, the discriminant functions were named based on their most distinctive aspects: Function 1 as “*Schizotypal variate*” and Function 2 as “*Narcissistic, antisocial, histrionic variate*”.^3^ The discriminant plot and group centroids showed that the first function, to which the schizotypal dimension (*r* = .924) was most strongly correlated, discriminated the *Schizotypal/paranoid* cluster from the other clusters. The second function to which the narcissistic (*r* = .526), antisocial (*r* = .324) and histrionic PD dimensions (*r* = .311) were highly correlated, discriminated the second *Extravert/externalizing* cluster from the other two clusters.

### Validation of the clusters

#### Demographic and clinical features

The *Extravert/externalizing* cluster was composed of significantly more men (37%, *n* = 10) than the *Core BPD* cluster (8%, *n* = 11) and the *Schizotypal/paranoid* cluster (13%, *n* = 2), *p* < .001 (Fisher’s exact test). Clusters did not differ significantly on other demographic characteristics, such as having daytime activities (χ^2^(2) = .59, *p* = .761), living environment (*p* = .991, Fisher’s exact test), or age (*F*(2, 184) = .76, *p* = .469).

The clusters differed significantly in number of axis-I disorders (*F*(2, 184) = 4.10, *p* = .018). Patients in the *Extravert/externalizing* cluster had significantly fewer axis-I disorders (*M* = 1.3, *SD* = 1.0) than those in the *Schizotypal/paranoid* (*M* = 2.5, *SD* = 1.2, *p* = .007) and *Core BPD* (*M* = 1.9, *SD* = 1.5, *p* = .027) clusters.

#### Validation measures

Table 3 shows cluster means and ES for the other validation measures. A trend on the MANOVA for borderline pathology (PAI-BOR, Λ = .91, *F*(8, 302) = 1.73, *p* = .091) was found. Follow-up ANOVAs showed a significant difference between the clusters for the total PAI-BOR score (*F*(2, 154) = 3.60, *p* = .030), which did not result in significant post-hoc comparisons. Significant differences between the clusters were found for the Affective Instability (*F*(2, 154) = 3.70, *p* = .027) and Identity Problems (*F*(2, 154) = 4.66, *p* = .011) subscales. The Games-Howell post-hoc test showed that patients in the *Extravert/externalizing* cluster tended to report less affective instability, but this trend did not reach significance (*p* = .065). However, *Extravert/externalizing* patients did report significantly less identity problems (*p* = .027) compared with those in the *Core BPD* cluster.

**Table 3:** Cluster means and effect sizes on validation measures

		Core BPD (*n* = 83–125)^a^		Extravert/externalizing (*n* = 11–19)^a^		Schizotypal/paranoid (*n* = 9–14)^a^		Total (*n* = 103–165)^a^		Cohen’s *d*		
		*M*	*SD*	*M*	*SD*	*M*	*SD*	*M*	*SD*	*1–2*	*1–3*	*2–3*
PAI-BOR	Total	48.45	9.51	42.95	11.62	43.46	11.74	47.37	10.13	.54	.49	.05
	Identity Problems	12.76	3.24	10.53	3.26	11.31	3.30	12.37	3.32	.67	.44	.24
	Affective Instability	13.66	2.71	11.79	3.26	12.85	3.95	13.37	2.94	.64	.28	.36
	Negative Relationships	12.68	2.95	11.68	3.30	12.00	3.14	12.50	3.01	.33	.23	.10
	Self-Harm	9.34	4.25	8.95	4.18	7.31	4.17	9.13	4.25	.09	.48	.39
BSI	Total	1.91	.76	1.49	.89	1.71	.90	1.84	.79	.53	.24	.28
IIP	Total	112.32	39.98	96.35	35.86	110.18	42.07	110.19	39.79	.40	.05	.35
	Domineering/Controlling	9.50	5.72	11.00	6.10	8.43	5.24	9.59	5.72	.26	.19	.45
	Vindictive/Self-Centered	11.75	5.53	11.68	6.42	13.07	6.16	11.86	5.67	.01	.23	.24
	Cold/Distant	13.21	7.41	12.84	7.09	13.21	6.39	13.17	7.24	.05	.00	.05
	Socially Inhibited*	16.24	8.45	11.56	6.57	16.21	5.91	15.66	8.16	.57	.00	.57
	Overly Accomodating	15.56	7.50	12.26	6.57	15.43	8.93	15.15	7.56	.44	.02	.42
	Non-Assertive*	16.36	7.84	10.63	6.18	17.53	8.26	15.76	7.90	.72	.15	.87
	Self-Sacrificing^b^	17.29	6.94	13.95	6.32	16.00	6.66	16.77	6.89	.48	.19	.30
	Intrusive/Needy	12.43	5.45	12.42	5.27	10.30	5.32	12.23	5.42	.00	.39	.39
EQ-5D	Total**	.48	.28	.65	.17	.60	.30	.51	.28	.59	.43	.16
	EQ-VAS	57.15	19.29	62.12	16.64	70.23	24.61	58.82	19.76	.25	.65	.40
SIPP	Self-Control	2.10	.60	2.29	.66	2.17	.55	2.12	.60	.32	.11	.20
	Identity Integration*	1.90	.60	2.42	.65	1.88	.62	1.95	.63	.83	.03	.86
	Responsibility	2.60	.57	2.52	.69	2.75	.47	2.60	.57	.13	.26	.39
	Relational Capacities	2.29	.64	2.50	.62	2.39	.43	2.32	.63	.34	.16	.18
	Social Concordance	2.78	.60	2.72	.66	2.83	.45	2.78	.59	.11	.08	.19
ECR	Anxiety	5.09	1.17	4.67	.96	5.17	.52	5.06	1.21	.38	.07	.45
	Avoidance	3.70	1.29	3.77	1.52	3.57	1.44	3.70	1.32	.05	.10	.15
CTQ	Total	54.78	19.09	61.09	22.00	56.22	19.75	55.58	19.36	.33	.07	.25
	Emotional Abuse^b^	14.22	6.02	17.55	6.04	15.22	6.87	14.66	6.12	.54	.16	.38
	Emotional Neglect	15.13	5.60	17.45	6.04	16.22	6.00	15.48	5.68	.41	.19	.22
	Physical Abuse	7.72	4.61	8.64	5.64	6.89	3.52	7.75	4.62	.20	.18	.37
	Physical Neglect	8.90	3.58	9.73	4.10	9.22	3.27	9.02	3.59	.23	.09	.14
	Sexual Abuse	8.81	5.55	7.73	5.97	8.67	4.90	8.68	5.50	.20	.03	.17

*PAI-BOR* Personality Assessment Inventory borderline features scale, *BSI* Brief Symptom Inventory, *EQ-5D* EuroQol EQ-5D-3 L, *SIPP* Severity Indices of Personality Problems, *ECR* Experiences in Close Relationships questionnaire, *CTQ* Childhood Trauma Questionnaire

^a^= n varies due to missing values. Cohen’s *d* columns show effect sizes between, respectively, clusters 1–2, 1–3, and 2–3. **Significant in (M)ANOVA at *p* < .05. *Marginally significant in (M)ANOVA with moderate to large ES

^b^Probable distinguishing based on moderate ES

A trend was found between the clusters on symptomatic severity (BSI; *F*(2, 162) = 2.56, *p* = .081), with patients in the *Core BPD* cluster reporting the highest symptomatic severity, and the *Schizotypal/paranoid* and the *Extravert/externalizing* clusters reporting below overall sample mean symptomatic severity.

No significant differences between the clusters were found in an overall MANOVA on interpersonal problems (IIP; Λ = .84, *F*(18, 290) = 1.45, *p* = .107). Separate ANOVAs for each subscale showed that the clusters differed only on the Socially Inhibited (Welch statistic, *p* = .032) and Non-Assertive (*F*(2, 153) = 4.95, *p* = .008) subscales. A Games-Howell post-hoc test showed that patients in the *Extravert/externalizing* cluster had significantly less interpersonal problems related to social inhibition than those in the *Core BPD* cluster (*p* = .026). The *Extravert/externalizing* cluster reported significantly less interpersonal problems related to nonassertiveness, in comparison to the elevated scores for non-assertiveness in the other two clusters.

A significant difference was found between the clusters for quality of life (EQ), Λ = .94, *F*(4, 298) = 2.56, *p* = .039. A follow-up ANOVA showed a significant difference between the clusters on the EQ score (*p* = .021) by means of the Welch statistic. Patients in the *Core BPD* cluster reported the lowest quality of life and scored significantly lower than patients in the *Extravert/externalizing* cluster, who reported the highest quality of life (*p* = .006). A trend was found for the EQ VAS score (*F*(2, 151) = 2.50, *p* = .085), with the *Schizotypal/paranoid* cluster having highest self-reported health, followed by the *Extravert/externalizing* cluster and then the *Core BPD* cluster, which reported below the (overall) mean state of health, although post-hoc tests did not reach significance.

No significant differences were found on personality functioning (SIPP; Λ = .91, *F*(10, 300), =1.54, *p* = .124), attachment (attachment avoidance; *F*(2, 141) = .075, *p* = .928; attachment anxiety, Welch statistic *p* = .258), or trauma history (CTQ; Λ = .945, *F*(10, 192) = .553, *p* = .850).

**Fig. 2:**
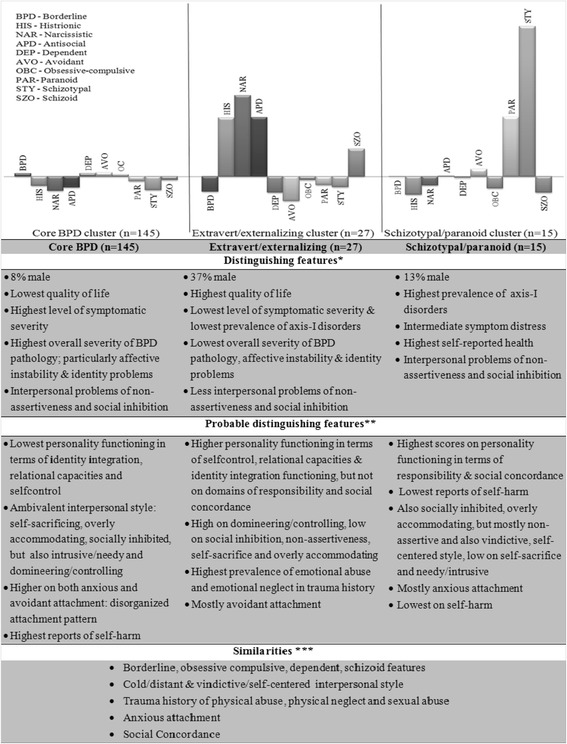
Distinguishing, probable distinguishing features and similarities of the clusters. All features are relative compared to the other clusters (as oposed to norm groups). *Significant distinguishing based on significant ANOVA. ** Probable distinguishing based on moderate (> .5) or large ES (> .8). *** Similarities based on small ES (< .2)

Moderate ES (see also Table 3) represented differences between the clusters in terms of symptomatic severity, quality of life, attachment style (specifically in terms of attachment anxiety), emotional abuse, and emotional neglect, and overall severity of borderline pathology, as well as affective instability and identity problems. The latter finding is confirmed by a large ES on Identity Integration. In order to enhance interpretability, the directions of the differences based on ES are summarized as (potentially) distinguishing features between the clusters in Fig. 2, which also presents the similarities between the clusters.

## Discussion

Results of this study showed three meaningful clusters of BPD patients with distinctive profiles, suggesting three potential subtypes of BPD: (a) a *Core BPD*, (b) an *Extravert/externalizing*, and (c) a *Schizotypal/paranoid* subtype. The subtypes were clearly gendered, in that men were remarkably more prevalent within the *Extravert/externalizing* subtype compared with the total sample and the other subtypes. In addition, subtypes differed in terms of quality of life and number of comorbid symptoms, with the *Extravert/externalizing* subtype reporting the highest quality of life and lowest number of axis-I disorders. Trends were found for domains of interpersonal problems and borderline pathology severity, the latter specifically in terms of affective dysregulation and identity problems, with the *Extravert/externalizing* subtype again reporting the least problems in these domains. Probable distinguishing features between the subtypes included specific aspects of personality functioning and attachment. The remarkable differences in the number of patients per cluster, with the *Core BPD* cluster containing five times as many patients as the *Extravert/externalizing* cluster and almost 10 times as many patients as the *Schizotypal/paranoid* cluster may be an important finding in itself. However, further replication of this finding is needed, as it may be influenced by the specific treatment setting, and/or the results may indicate that the *Extravert/externalizing* and S*chizotypal/paranoid* patients are less inclined to seek treatment.

The *Core BPD* subtype had relatively more BPD features, but was mainly characterized by the absence of marked elevation on any of the other PD dimensions. This subtype resembles the nonaggressiveness/nonparanoid/nonantisocial subtype of Lenzenweger et al. [14] and the avoidant/obsessive-compulsive subtype reported by Critchfield et al. [32], in terms of the similar presence of avoidant features, and the anaclitic BPD type reported by Blatt and Auerbach [68]. The *Core BPD* subtype appears to represent the prototypical BPD patients for whom most evidence-based treatments seem to be initially developed. These patients generally reported the highest symptomatic severity and personality pathology. This group did not clearly differentiate on either attachment avoidance or attachment anxiety, which might suggest a disorganized attachment style. Their pattern of interpersonal problems showed ambivalence in terms of an internalizing style characterized by social inhibition, nonassertiveness, and being overly accommodating on the one hand, while, on the other hand, having a high need for closeness and an intrusive, dominant, controlling style. The contradictory phenomenon of the high need for intimacy and simultaneous experience of high anxiety in response to intimacy is often observed clinically in these patients. In accordance with this, *Core BPD* patients reported marked relational problems, but also instability in identity and self-control. Remarkably, though, these patients reported the lowest levels of early childhood trauma. This contrasts with theories that suggest an important role for environmental adversity, but supports theories that point to the importance of biological vulnerability in the etiology of BPD [7, 69]. However, because levels of trauma were overall high in the total sample, it should be borne in mind that differences between the subtypes are relative, and that ES of differences in trauma between subtypes were small for most categories of trauma. Moreover, Weinstein et al. [70] have pointed to the importance of focusing on specific types of childhood trauma. Although different forms of trauma were assessed in this study, the evaluation of childhood trauma was not exhaustively assessed (i.e., there was a lack of assessment of more subtle forms of unavailability of the caregiver who has the child in mind, which has previously been mentioned as an important influential factor in the etiology of BPD pathology; [7]). Moreover, possible confounding of the concept of dissociation in the relationship between trauma and BPD pathology [70] was not accounted for in this study, and this might have influenced our findings.

The *Extravert/externalizing* subtype was labeled as such because an outward-oriented/externalizing attitude seemed to be a common denominator in the narcissistic, antisocial, and histrionic PD dimensions on which this subtype differentiated from the other two subtypes. Correspondingly, this subtype scored very low on the avoidant PD dimension, in contrast to the other two subtypes. This subtype resembles similar subtypes of patients found by Critchfield et al. [32] and Lenzenweger et al. [14] (i.e., labeled narcissistic and histrionic, and antisocial/aggressive/nonparanoid, respectively) and the introjective BPD type reported by Blatt and Auerbach [68]. These patients reported relatively low symptomatic severity and generally more adaptive levels of personality functioning, with their interpersonal functioning being characterized by a dominant, self-centered style. It could be hypothesized that these externalizing patients have a tendency to deny distress and/or may experience less problems or burden. Caligor, Kernberg, and Clarkin [71] have described such a subtype of patients that seems to be able to function relatively stable in certain domains. Nevertheless, it could be expected that these patients have problems adjusting to social norms, as a result of their externalizing style. This was confirmed by impairments on domains of responsibility and social concordance, which cover aspects of personality functioning such as responsibility, trustworthiness, respect, and cooperation. These patients are often known for their high levels of dismissive attachment and indeed scored higher on attachment avoidance compared to the other subtypes. Perhaps surprisingly, this subtype reported more childhood trauma (especially emotional abuse and emotional neglect) compared with the other subtypes; this might reflect a relatively more important role for environmental factors in the etiology of BPD in these patients [7]. Men were overrepresented in this subtype, which might indicate a gendered expression of BPD pathology, marked by a tendency for men to externalize problems. However, results may also indicate that internalizing male BPD patients are less likely to seek treatment or to be referred for treatment. The subtype of patients with BPD and comorbid narcissistic and antisocial PD features has previously been identified as a group that poses significant clinical challenges and might be more treatment resistant [72], necessitating a somewhat different treatment approach (i.e., [71]). Indeed, the developers of several current evidence-based treatments initially developed for BPD have adapted their core models and treatment programs for this subtype [72–74].

The third subtype, *Schizotypal/paranoid*, was labeled as such because of the elevated levels of schizotypal and, to lesser extent, paranoid features that were evident. This subtype appears quite similar to the cluster A subtype with elevated schizotypal paranoid features identified by Critchfield et al. [32], and to the paranoid/nonaggressive/nonantisocial group reported by Lenzenweger et al. [14]. Questions arise whether this subtype may (partly) represent a group of patients with higher risk for psychosis, as schizotypy has been described as being associated with developing psychotic spectrum disorder [75]. The group shares some commonalities with the borderline schizophrenia subtype reported by Blatt and Auerbach [68]; however, these authors mention that patients meeting criteria for both BPD and schizotypal PD (as is the case in this subtype) are most likely to be introjective individuals, who are differentiated from the borderline schizophrenia subtype by having more intact perceptual processes and less vulnerability to fully psychotic states (although transient, reversible psychotic regressions may be present in introjective patients). Badoud al. [76] also underlined the fact that similar symptoms (such as psychotic manifestations) may be different in nature and duration when occurring in the context of borderline pathology versus schizotypal pathology, and may need a different treatment approach. The fact that a separate subtype emerged with marked elevations on this dimension underlines the importance of investigating this trait within BPD patients. Despite the fact that co-occurrence of schizotypal traits and BPD is widely documented, and is generally considered to be challenging to treat [76], current treatment manuals do not propose specific adaptations to their approaches for these patients. This subtype might be less inclined to seek treatment or less able to get into treatment, which may also in part explain why this cluster was so small. Patients within this subtype report relatively more stable functioning in terms of responsibility and social concordance and higher self-reported health. This may be accounted for by the fact that these patients seem to function in a socially isolated way, resulting in them experiencing less distress. Although problems occur in interpersonal relationships related to social inhibition and being overly accommodating, similar to the *Core BPD* subtype, the *Schizotypal/paranoid* subtype does not show the same ambivalence, and there is no intrusive interpersonal style in need for closeness, as is the case in the *Core BPD* subtype. In contrast, this subtype shows more problems concerning hostile dominance, which is marked by mistrust. They also exhibit lower levels of self-sacrifice in comparison to the *Core BPD* subtype, but show a lack of self-confidence and problems with assertiveness. The *Schizotypal/paranoid* subtype showed highest levels of attachment anxiety compared to the other subtypes.

As noted above, the subtypes found in our study resemble subtypes that were reported by Critchfield et al. [32] and Lenzenweger et al. [14], probably due to the fact that the clustering procedure was based on similar clustering dimensions. Comparison with other studies is hampered by dissimilarity in the characteristics on which subtypes were formed and described, as well as in the validation measures used. Yet, an externalizing subtype similar to our *Extravert/externalizing* subtype has been delineated in multiple studies: an angry externalizing and histrionic subtype (both loading heavily on the externalizing dimension) in the adolescent study of by Bradley et al. [29], an externalizing dysregulated subtype in an adult sample [28], and an anxious externalizing subtype [30]. Nearly all previous studies also describe subtypes that in some way resemble our *Core BPD* and *Schizotypal/paranoid* subtypes; however, there is considerable variability in terms of how these subtypes are defined and categorized. All these subtypes seem to share an internalizing stance compared with the externalizing subtypes mentioned above. This might suggest that an internalizing–externalizing dimension is important in understanding the heterogeneity of BPD, as has been previously suggested [31, 77].

Importantly, while the subtypes showed distinct features on both the PD comorbidity profiles and relevant concepts such as symptomatic severity, attachment dimensions, identity problems, affective instability, quality of life, and interpersonal functioning, differences were small and the subtypes also showed similarities. Interpersonal problems related to a cold/distant and self-centered attitude, attachment avoidance, trauma history of physical abuse, physical neglect and sexual abuse, and personality functioning in terms of social concordance were all domains that showed similarity rather than differentiation between the subtypes. These shared features might be accounted for by the fact that the sample comprised a treatment-seeking population with overall high levels of psychopathology. Correlations between the PD dimensions that remained high in spite of correcting for overall severity indicated the presence of comorbidity that cannot merely be explained by severity in terms of overall personality pathology. A “general p factor” has been identified by Caspi and colleagues [78, 79], which may underlie severe psychopathology and might be responsible for commonalities across the subtypes. On the other hand, Caspi et al. [78] mention an internalizing, externalizing, and thought disorder dimension as accounting for individual differences in symptom picture, although these do not explain harmful dysfunction net of the p factor. These dimensions might explain the differences that were found in phenotypic expression, aside from the common general psychopathology. Accordingly, two discriminant dimensions were shown to account for the differentiation of the subtypes in this study. The *Narcissistic, antisocial, histrionic* variate and the *Schizotypal* variate resemble to some extent the underlying “internalizing/externalizing” and “thought disorder” factors that have repeatedly been demonstrated in studies on the underlying dimensions of psychiatric comorbidity [78, 80, 81].

Future research should extend the current findings by the use of underlying dimensions instead of classification-based data. This might lead to improved understanding of how common-ground dimensional factors such as internalizing/externalizing, thought disorder and overall psychopathology differentiate meaningful subtypes of BPD. This corresponds with the suggestion of Fossati et al. [21] that more meaningful subtypes might be found when taking more dynamic, developmental, dimensional factors into consideration. Future research should also include the notion of differing etiological pathways by examining biomarkers in identified subtypes, as well as including measures that facilitate the identification of working mechanisms. The current findings merely pose a first, though important, step in the possible refinement of therapies and the improvement of treatment outcome, as well as an improved understanding of empirical research on BPD. The results enable researchers to easily categorize patients within the distinct subtypes both within a clinical and research context, based upon their comorbidity profile. The next step would be to start using the identified subtypes in continued research efforts, in order to check for robustness but also to prove their surplus value in research on treatment outcome, trajectories of response, working mechanisms as well as studies on etiology of BPD. Furthermore, as mentioned existing treatment programs are likely tailored for the vast majority and lack specific elements necessary to deal with the characteristics (either full-blown or subthreshold comorbid PD pathology) belonging to the *Externalizing* and the *Schizotypal/paranoid* subtype. Future research should take the course of investigating whether or not existing adaptations of evidence based treatment programs [72–74] promote treatment success specifically within these subtypes. At the same time the dearth of treatment programs for these latter two subtypes calls for further innovation of existing treatment programs to tailor to the specific needs of these subtypes. Moreover, to target the *Schizotypal/paranoid* subtype specifically, there is a challenge in reaching out to these potentially treatment refractory patients, as the results suggest that they may be less inclined to seek help.

Most importantly, the current results raise important questions about the implications of the observed BPD subtypes for research on treatment outcome in terms of (cost-)effectiveness and treatment trajectories, with implications for treatment indication and tailored interventions during treatment. To the best of our knowledge, only Digre et al. [30] have studied subtypes of BPD patients in the context of differential treatment outcome. In that study, three subtypes of BPD patients (withdrawn-internalizing, severely disturbed-internalizing, and anxious-externalizing) were found. The withdrawn-internalizing subtype improved in terms of reduced levels of dissociation, while treatment resolved primarily depressive symptoms in the anxious-externalizing subtype. The severely disturbed-internalizing subtype, which shows some resemblance to both our *Core BPD* and our *Schizotypal/paranoid* subtype, did not improve significantly on any outcome measure. A follow-up study will examine treatment trajectories of the subtypes identified in this study in the context of a multi-site trial on the efficacy and cost-effectiveness of MBT [2].

Although the study included a relatively large overall sample, its statistical power to find differences was rather limited due to the fact that we allowed the sample sizes of the different subtypes to be unequal in order to amplify the external generalizability of the results based on the idea that the prevalence of distinctive profiles is likely to differ in clinical practice. This resulted in two small clusters and consequently a relative lack of statistical power in comparing the clusters on validation measures. Furthermore, several limitations concerning the characteristics of the present sample dictate caution in interpreting the results of this study. Although generalizability was maximized by using few exclusion criteria, generalizability to BPD in general is somewhat doubtful, because the sample included only treatment-seeking patients. In addition, although comorbidity in terms of traits was high, this was not the case in terms of PD diagnosis. Moreover, our sample included mostly female patients, whereas there is no evidence of BPD being more common in women [1]. Although the gender differences between subtypes were profound and some hypotheses explaining this finding have been mentioned above, solid interpretation is hampered by the fact that the distribution of male versus female patients in the overall sample was uneven. Hence, further research is needed to replicate these findings in other samples.

## Conclusion

In sum, this study found three meaningful subtypes that are roughly in line with previous reports and show clinical differences on validation measures. Common underlying factors such as p might account for the similarities, while underlying dimensional constructs also seem to account for the subtype distinction. This parallels the clinical impression that, although they share common features and severity of pathology, patients present with different clinical presentations. The results may be a stepping stone toward research focusing on differential trajectories of change, treatment outcome, and treatment approaches for these distinct subtypes.

## Abbreviations

ANOVA: Univariate analysis of variance
BPD: Borderline personality disorder
BSI: Brief symptom inventory
CTQ: Childhood trauma questionnaire
DSM: Diagnostic and statistical manual of mental disorders
ECR: Experiences in close relationships
EQ: EuroQol
ES: Effect size
IIP: Inventory of interpersonal problems
MANOVA: Multivariate analysis of variance
MBT: Mentalisation-based treatment
PAI-BOR: Personality assessment inventory-borderline
PD: Personality disorder
SCID-I: Structured clinical interview for DSM-IV axis I personality disorders
SCID-II: Structured clinical interview for DSM-IV axis II personality disorders
SD: Standard deviation
SIPP: Severity indices of personality problems
SPSS: Statistical package for the social sciences
VAS: Visual analogue scale

## Footnotes

1 For three patients who did not complete the SCID-I, Axis I diagnoses were based on the diagnosis at intake established by an experienced MSc-level clinical psychologist or psychotherapist.

2 Correlations between personality dimensions corrected and uncorrected for severity may be obtained from the first author.

3 Correlations between personality dimensions and canonical discriminant functions are available on request from the first author.
